# Dosimetric and geometric evaluation of the use of deformable image registration in adaptive intensity-modulated radiotherapy for head-and-neck cancer

**DOI:** 10.1093/jrr/rru044

**Published:** 2014-06-06

**Authors:** R.B. Eiland, C. Maare, D. Sjöström, E. Samsøe, C.F. Behrens

**Affiliations:** Department of Oncology, Radiotherapy Research Unit, Herlev Hospital, University of Copenhagen, Herlev Ringvej 75, DK2730 Herlev, Denmark

**Keywords:** adaptive radiotherapy, deformable image registration, head and neck

## Abstract

The aim of this study was to carry out geometric and dosimetric evaluation of the usefulness of a deformable image registration algorithm utilized for adaptive head-and-neck intensity-modulated radiotherapy. Data consisted of seven patients, each with a planning CT (pCT), a rescanning CT (ReCT) and a cone beam CT (CBCT). The CBCT was acquired on the same day (±1 d) as the ReCT (i.e. at Fraction 17, 18, 23, 24 or 29). The ReCT served as ground truth. A deformed CT (dCT) with structures was created by deforming the pCT to the CBCT. The geometrical comparison was based on the volumes of the deformed, and the manually delineated structures on the ReCT. Likewise, the center of mass shift (CMS) and the Dice similarity coefficient were determined. The dosimetric comparison was performed by recalculating the initial treatment plan on the dCT and the ReCT. Dose–volume histogram (DVH) points and a range of conformity measures were used for the evaluation. We found a significant difference in the median volume of the dCT relative to that of the ReCT. Median CMS values were ∼2–5 mm, except for the spinal cord, where the median CMS was 8 mm. Dosimetric evaluation of target structures revealed small differences, while larger differences were observed for organs at risk. The deformed structures cannot fully replace manually delineated structures. Based on both geometrical and dosimetrical measures, there is a tendency for the dCT to overestimate the need for replanning, compared with the ReCT.

## INTRODUCTION

Intensity-modulated radiotherapy (IMRT) uses steep dose gradients and thus requires accurate patient positioning [1–4]. Anatomical changes, as a result of e.g. weight loss or tumor shrinkage, may occur during the course of treatment. For head-and-neck cancer patients, several studies have shown that these changes potentially lead to a significant difference between planned and delivered doses [5–7]. If the anatomical changes cannot be accounted for by adjusting the patient position, an adaptation of the treatment may be necessary [8–10]. However, this can be a very time-consuming process because it often requires a new CT scan and re-delineation of structures. For head-and-neck cancer patients with many targets and organs at risk (OARs), this procedure may take several hours [11]. Deformable image registration (DIR) has the potential to ease this process by deforming the contours from the planning CT (pCT) to the new anatomy of the patient represented by a rescanning CT (ReCT) or a cone-beam CT (CBCT) [8, 12–17]. The aim of this study was to perform geometric and dosimetric evaluation of the usefulness of a commercially available DIR algorithm utilized for adaptive head-and-neck IMRT [18].

## MATERIALS AND METHODS

For all patients, a deformation vector field was obtained by deforming the pCT to match a CBCT scan acquired approximately halfway through the course of treatment. The structures manually delineated on the pCT were deformed using the deformation vector field. Structures manually delineated on an ReCT, acquired the same day (±1 d) as the CBCT, served as ground truth for evaluation of the deformed structures. All CT and CBCT scans were obtained as part of clinical practice, and this retrospective study was undertaken as a quality assurance of this practice. In Denmark, such studies do not need Institutional Review Board approval by the Ethics Committee (The National Committee on Health Research Ethics).

### Patient data

This study included data from seven head-and-neck cancer patients (Table 1). Both the pCT and the ReCT had a corresponding structure set containing structures manually delineated by the same physician((C.M.) (who also manually delineated/created the CTV on a deformed CT (dCT), as explained below). Interobserver variations were excluded by letting the same physician perform all delineations. It was not possible to exclude intraobserver variation. The IMRT plans used for treating the patients were available on pCT. In accordance with national guidelines, the plans were constructed aiming for 95–107% of the prescribed dose to at least 99% of the PTV, whereas 1% of the PTV was allowed to receive 90–95% of the prescribed dose. The plans were normalized in such a way that the mean dose to the PTV equaled the prescribed dose. The dose calculation was carried out using the anisotropic analytical dose calculation algorithm in the treatment-planning system (TPS) Eclipse (Varian Medical Systems, v.10.0). All treatments were performed on a Varian Clinac iX [19], and CBCT scans were conducted with the ‘Standard Dose Head’ protocol.

**Table 1.** Patient data

Patient	Primary	Dose	Fractions	CBCT	ReCT
ID	site	(Gy)		(Frac)	(Frac)
1	Unknown	68	34	17	18
2	Oropharynx	68	34	17	17
3	Cavum oris	68	34	17	17
4	Oropharynx	68	34	18	18
5	Oropharynx	66	33	29	29
6	Oropharynx	68	34	24	24
7	Oropharynx	68	34	23	22

Dose = prescribed dose, Fractions = total number of treatment fractions, CBCT [Frac] = fraction at which the CBCT was acquired, ReCT [Frac] = fraction at which the ReCT was acquired.

### Deformable registration and contour propagation

The DIR and contour propagation was performed in *SmartAdapt®* (Varian Medical Systems ver. 11.0). The DIR is based on a Demons algorithm [18, 20]. Initially, a rigid registration followed by DIR was performed between the pCT and the CBCT. It was visually checked that the rigid registration was ‘as good as possible’, which basically means that the bones aligned well. There will always be small differences in set-up between different scans, and perfect alignment is not possible. This fact was ignored in the data analysis but is considered in the Discussion. The CBCT had a scan range smaller than the pCT, and DIR was only performed within the dimensions of the CBCT. The DIR produced a new image set (dCT) with the image quality of the CT and the anatomical information of the CBCT. After the DIR, the structures from the pCT were deformed and propagated to the dCT. The CTV is based on the clinical knowledge of the radiation oncologist [21]. However, from the DIR the structure is based on image intensity. Therefore, manual delineation/creation of the CTV was conducted by C.M., based on the geometric expansion of the deformed GTV. This followed the same procedure as carried out for the clinically used treatment plan. As a first step to create the CTV-T (tumor) and CTV-N (node), a 10 mm margin was added to the deformed GTV. From this, the final CTV was created by removing all extensions into air cavities and bones together with extensions outside the patient outline. Finally, the planning target volume (PTV) was created by adding a 5 mm margin to the CTV. Note that, even though the CTV and PTV were manually delineated/created on the dCT, they reflect the performance of the DIR because they are based on the deformed GTV.

### Geometrical comparison

The volumes (V_ReCT_s) of the manually delineated structures on the ReCT, and the volumes (V_dCT_s) of the structures on the dCT were recorded and evaluated. Because the DIR was only performed within the volume of the CBCT, a body outline corresponding to the CBCT was created. Structures reaching outside this volume were cropped to the body outline of the CBCT such that a common ground for comparison was created. The center of mass shift (CMS) and Dice similarity coefficient (DSC) were used to compare the structures using the ReCT as the ground truth. DSC was defined as:

(1) DSC=2(VReCT∩VdCT)VReCT+VdCT.

### Dosimetric comparison

The pCT treatment plan was transferred to the ReCT and the dCT, and calculation of dose was carried out with the same settings, including the same number of monitor units. To compare the plans recalculated on the dCT and the ReCT, DVH points where determined. In order to evaluate the dose coverage of the GTV and the CTV, the D_median_, D_98%_ and D_2%_ were determined [21]. The dose to the spinal cord and the dose to the parotid glands were determined by D_max_ and D_mean_, respectively, according to the radiotherapeutic guidelines from the Danish Head and Neck Cancer Group (DAHANCA). In addition to the DVH points, the lesion coverage fraction (LCF) and the normal tissue overdosage fraction (NTOF) were determined, indicating plan conformity. LCF is an expression for the fraction of the PTV covered by the 95% isodose line. To find the fraction of the PTV covered by the 95% isodose, a Boolean operator available in the TPS was used to include all pixels belonging to both the PTV structure and the 95% isodose structure (V_PTVand95_). In cases where the patient had two or more defined PTVs, these were joined in one structure for the calculation of LCF and NTOF [22, 23]. LCF is defined as:

(2) $$\mathrm{LCF}=\frac{V_{\mathrm{PTVand}95}}{V_{\mathrm{PTV}}},$$

where V_PTV_ is the volume of the PTV. If the PTV is completely included in the 95% isodose, the LCF will equal unity [22]. NTOF is an expression for the fraction of the 95% isodose delivered to normal tissue and is defined as:

(3) $$\mathrm{NTOF}=\frac{V_{\mathrm{PTVsub}95}}{V_{95}},$$

where V_95subPTV_ is the volume of the 95% isodose structure with all pixels included in the PTV subtracted, and V_95_ is the volume covered by the 95% isodose. Ideally, NTOF will equal zero, indicating that no normal tissue is inside the 95% isodose [22].

## RESULTS

### Geometrical comparison

**Fig. 1.**
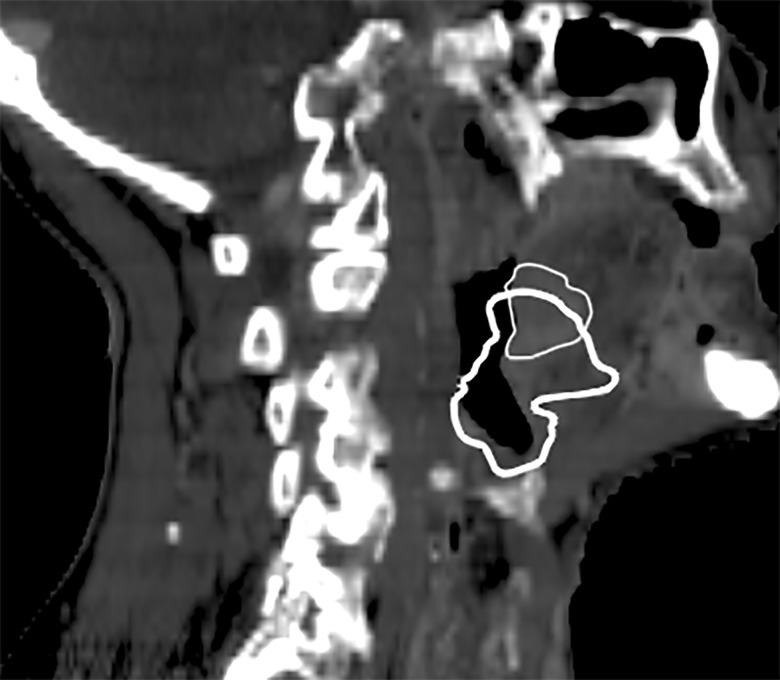
Sagittal image of the patient excluded from the GTV-T analysis due to large differences between the GTV-T from the pCT and the ReCT. The GTV-T from the dCT (bold line) and the ReCT (thin line) are shown on the ReCT scan.

All results are presented as median values and ranges. For the GTV-T, structures for five patients are presented (Table 2). One of the patients only had a GTV-N. A second patient experienced large anatomical changes in the GTV-T, and the changes as recorded by the physician on the ReCT differed considerably compared with the DIR (on dCT) (Fig. 1). To investigate this further, the physician (C.M.) inspected the delineations on both the pCT and the ReCT and concluded that the delineations were correct. Hence, the differences between the delineations on the pCT and the ReCT were due to actual anatomical changes within the patient. Evaluation of the deformed image (not shown) revealed a narrower air cavity, which partly explains the difference between the two structures. Since this patient data would have a large influence on the overall results for the GTV-T, it was excluded as an outlier in the GTV-T analysis. The DIR did not systematically produce either larger or smaller volumes on the dCT, compared with the manual delineation on the ReCT (Table 2). For the target structures, the CMS was within a similar range for both the nodes and the tumor, and it was up to more than 1 cm. The DSC values for the target structures revealed more similarity for the CTVs than for the GTVs, which is expected, simply because the volumes of the CTVs are larger. In most cases, the OARs were estimated to be smaller by the DIR than by manual delineation. Among the OARs, the largest median value of the CMS was found for the spinal cord, which also yielded the lowest median value of the DSC.

**Table 2.** Geometrical results presented as median value (range) of the differences between dCT and ReCT

Structure (number of patients)	Relative volume difference expressed as a percentage	CMS in cm	DSC expressed as a percentage
GTV-T (5)	23.5 (−10.8–69.0)	0.32 (0.13–0.88)	0.68 (0.54–0.78)
CTV-T (5)	−6,3 (−51.2–40.3)	0.49 (0.13–1.30)	0.80 (0.71–0.86)
GTV-N dxt (4)	23.8 (10.3–33.3)	0.27 (0.23–0.35)	0.74 (0.59–0.80)
CTV-N dxt (4)	11.0 (8.7–188.3)	0.21 (0.12–0.26)	0.88 (0.50–0.91)
GTV-N sin (4)	7.1 (−2.7–37.5)	0.33 (0.26–0.56)	0.54 (0.44–0.82)
CTV-N sin (4)	6.5 (−7.4–148.2)	0.37 (0.29–0.60)	0.80 (0.57–0.85)
Parotid dxt (7)	7.9 (−27.0–38.1)	0.22 (0.14–0.45)	0.78 (0.73–0.83)
Parotid sin (7)	−8.9 (−22.3–9.4)	0.24 (0.18–0.68)	0.81 (0.68–0.85)
Spinal cord (7)	−22.3 (−28.9–−7.9)	0.80 (0.25–1.31)	0.67 (0.64–0.73)

The relative volume difference is relative to the ReCT.

### Dosimetric comparison

**Fig. 2.**
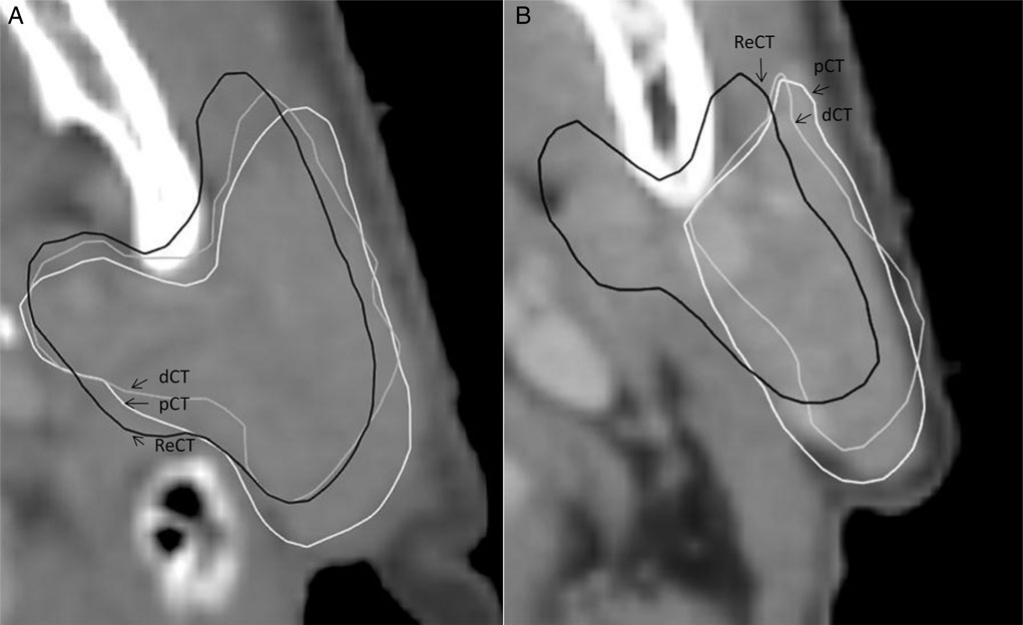
Visualization on the pCT of the patient with the lowest value of DSC for the parotid gland in two different CT slices. In some slices (**A**) the structures are similar, whereas large differences are observed between the dCT and the ReCT structures in other slices (**B**). The dCT structure resembles the pCT structure, and the large difference between the parotid on the pCT/dCT and the ReCT can, in part, be explained by intraobserver variations in the delineation.

For the target structures, the median percentage difference in DVH points was within 1.3%, indicating similar target coverage for all scans (Table 3). However, differences of up to 4.5% on the dCT (relative to the pCT) were observed. Evaluation of the DVH points for the parotid glands showed a larger median percentage difference. Figure 2 shows the largest observed difference between the delineation of the parotid gland for the ReCT and the dCT (i.e. for the patient with the smallest DSC for the parotid). In addition to the DVH points, the LCF and the NTOF were determined in order to evaluate the target coverage and the dose to normal tissues (Table 4). The LCF should ideally be equal to one, which was also the case for the median value for the pCT. The median LCF values for the dCT and the ReCT were lower than for the pCT. However, values of the pCT and the ReCT were similar, indicating similar target coverage. For two patients, the LCF(dCT) equaled 0.97, while the LCF(ReCT) equaled 0.95; for the remaining patients, the LCF(dCT) was less than the LCF(ReCT). Lower values of the NTOF indicate that less normal tissue is inside the 95% isodose. The lowest median value of the NTOF was observed for the pCT. The median value of the NTOF was higher for the dCT and the ReCT, indicating that more normal tissue is included in the 95% isodose. The highest value was obtained for the ReCT.

**Table 3.** Difference in the DVH points relative to the pCT presented as median (range)

Structure (number of patients)	DVH point	dCT expressed as a percentage	ReCT expressed as a percentage
GTV-T (5)	D_median_	0.3 (0.1–2.4)	0.3 (0.0–1.0)
	D_98%_	−0.1 (−0.2–0.2)	0.1 (−0.8–1.4)
	D_2%_	0.0 (−0.3–0.2)	0.0 (0.0–0.0)
GTV-N sin (4)	D_median_	0.0 (0.0–0.0)	0.7 (0.2–1.0)
	D_98%_	−1.3 (−3.8–1.5)	−0.1 (−1.7–1.7)
	D_2%_	0.9 (−3.8–1.5)	0.7 (−0.2–1.2)
GTV-N dxt (4)	D_median_	0.6 (−2.6–0.8)	0.9 (0.8–1.0)
	D_98%_	1.0 (0.2–1.2)	1.0 (0.6–2.2)
	D_2%_	1.3 (−0.5–4.5)	1.0 (0.6–1.6)
CTV-T (5)	D_median_	0.5 (0.4–0.5)	0.4 (0.3–0.8)
	D_98%_	−0.1 (−0.2–0.2)	−0.1 (−0.2–0.2)
	D_2%_	0.4 (0.2–1.2)	0.5 (0.2–1.3)
CTV-N sin (4)	D_median_	0.2 (−0.1–3.8)	−0.1 (−0.2–1.4)
	D_98%_	−1.0 (−3.5–0.9)	0.0 (−0.2–0.0)
	D_2%_	1.1 (0.7–3.9)	0.8 (0.0–1.5)
CTV-N dxt (4)	D_median_	0.6 (−0.1–0.9)	1.0 (0.4–1.2)
	D_98%_	0.7(−3.8–0.9)	0.8 (−4.1–1.5)
	D_2%_	0.8 (0.7–2.1)	1.3 (0.7–1.8)
Parotid dxt (7)	D_mean_	9.4 (3.1–44.4)	−0.9 (−14.7–15.0)
Parotid sin (7)	D_mean_	5.6 (0.0–22.9)	0.0 (−14.4–32.3)
Spinal cord (7)	D_max_	0.2 (−2.9–26.7)	0.3 (−1.6–5.3)

**Table 4.** Conformity measures presented as median value (range)

Conformity measure	pCT expressed as a percentage	dCT expressed as a percentage	ReCT expressed as a percentage
LCF	1.00 (0.95–1.00)	0.93 (0.87–0.97)	0.96 (0.94–1.00)
NTOF	0.19 (0.04–0.20)	0.23 (0.19–0.38)	0.32 (0.13–0.49)

## DISCUSSION

The results suggest that for the DIR algorithm utilized in this study, the propagated deformed structures are not able to replace manual delineation completely. However, they could be used for assistance during the delineation on the ReCT, as proposed by Chao *et al*. [11]. Furthermore, if the dCT can be used to evaluate whether there might be a need for replanning, this would be valuable. In that case, the dCT may function as a decision tool for whether to rescan the patient or not. For this to be implemented as a clinical routine, the dCT may be allowed to overestimate the need for replanning but it should not underestimate the need. The latter would lead to suboptimal treatment for some patients.

The DVH points of the parotid glands had different corresponding values for the dCT and the ReCT, indicating that the positions of the structures in relation to the plan were significantly different. Difference in DVH points between the dCT and the ReCT may in part be explained by intraobserver variations in the delineations. However, intraobserver variation does not explain the difference in DVH points between the pCT and the dCT, which may have been caused by weight loss and tumor shrinkage shifting the parotids into a nearby area of higher dose. There was a tendency for the dosimetric changes for the OAR to appear worse on the dCT than on the ReCT, indicating that the dCT overestimated the need for replanning. As evaluated on the ReCT, most DVH points were within 2% of the pCT. This indicates the robust design of the treatment plan, i.e. yielding sufficient target coverage despite the observed anatomical changes. However, when evaluated from the dCT, larger differences of up to 4.5% were found. Similarly, the values for the LCF obtained from the dCT were generally lower than, or very close to, those obtained from the ReCT. Only in two patients was the LCF obtained from the dCT slightly higher (∼2%) than the LCF obtained from the ReCT. Again, if the dCT is used as a decision tool for when to consider replanning this is acceptable, since it will generally overestimate the need for replanning.

The most optimal median values of the LCF and the NTOF were obtained for the pCT. This was expected because the plan was optimized to the structures of the pCT. Values of the NTOF were found to be higher for the ReCT and the dCT compared with the pCT, indicating that more normal tissue was being included in the high-dose area. This is comparable with studies that have suggested that the primary benefit of replanning is in the sparing of normal tissue [24]. It should be noted that this would depend on the treatment technique. The IMRT plans evaluated in this study are robust to geometrical changes when it comes to target coverage. The same may not be true for other treatment techniques. The NTOF values tended to be higher on the ReCT than on the dCT, which indicates that using this parameter may lead to an undesirable underestimation of the need for replanning. If the patient set-up is well reproduced, the spinal cord is not expected to experience a large CMS or significant changes in volume. However, for two patients, a CMS of 1.31 cm was recorded between the dCT and the ReCT. These results were caused by a combination of the DIR not being able to fully compensate for differences in set-up between the pCT and the CBCT and intraobserver variation in delineations. Inadequate quality of the most caudal part of the CBCT probably also contributed to causing the DIR to deform the spinal cord incorrectly. For both patients with large CMSs, the most caudal part of the CBCT was noisy, resulting in a blurred appearance of the spinal canal. In this part of the CBCT, the deformed spinal cord was wider in the *x* and *y* directions compared with the spinal cord on the ReCT. This issue, combined with a too narrowly deformed spinal cord structure in the cranial part of the CBCT, caused a relatively large CMS in the *z* direction, i.e. in the caudal–cranial direction. Spinal cord volume changes of >20% were observed in four patients. In general, the width of the deformed spinal cord structures varied more along the *z* direction than the corresponding structures on the ReCT (or the pCT).

## CONCLUSION

In summary, there was a tendency for the dCT to overestimate the need for replanning when disregarding the NTOF parameter. Thus, the dCT could be used to evaluate whether a rescan was needed. Our geometric results suggest that automatically deformed structures, which are desirable for adaptive radiotherapy, are not accurate enough to replace manually delineated structures.

## FUNDING

Funding to pay the Open Access publication charges for this article was provided by the Department of Oncology, Herlev Hospital, University of Copenhagen.
